# Maternal Impressions and Fetal Malformations

**Published:** 1907-04

**Authors:** 


### MATERNAL IMPRESSIONS AND FETAL MALFORMATIONS.

---

As far back as authentic records extend and even beyond the time when historical fact fades out into myth and tradition, belief in the supernatural has possessed the mind of man. A concrete example of this is to be observed in the ancient theory of the effect of maternal impressions on the features. Even today when the light of scientific knowledge should illumine the minds at least of our writers on obstetrics, one finds here and there remnants if mediaeval superstition which modern reason has not suceeded in obliterating. One-half the text-books on Obstetrics written in America are unable to throw off this "sewing circle science" as one writer characterizes it.

It is apparently a fact that few call into question the theory of maternal impresions; they seem to consider it perfectly adequate to account for any and all physical and psychic defects. If one takes the trouble to ask questions, dozens of examples will be cited in which some shock or fright experienced by a pregnant woman is followed by the birth of a child with a defect, which by more or less stretching of the imagination is said to resemble the cause of the impression on the mother. They say the defect follows the maternal fright or shock, therefore it is the result of the impression on the maternal nervous mechanism. Herein lies the fallacy of the belief. Post hoc ergo propter hoc is the one argument upon which the theory rests, and to the lay mind this reasoning is entirely adequate. But here is where the reasoning falls down. No account is taken by the unscientific mind of the thousands of instances in which after a profound maternal shock during pregnancy no "marks" are found in spite of the fact that they are confidently expected. Again there are hundreds of cases of defect in the child in which the mother can recall absolutely no shock or impression to which the mark could be credited.

The maternal impressionist makes no attempt to account for the innumerable congenital anomalies and defects which occur in organs of which laymen have no knowledge. These are seen by thousands in dissecting rooms.

In the very lowest forms of life malformations are observed. "There is not a single malformation known to the human species that has not a corresponding malformation in the lower animals, both wild and domesticated. Analogous malformations also appear in the vegetable kingdom where single and double monsters abound, developments which result from arrested, defective or excessive formative energy." It is known that structural abberations originate very early in intrauterine development, not only, in many cases, long before the woman is aware of her condition but practically always long before the maternal mental impression to which the abnormality is ascribed takes place.

Moreover, it is well known that there is no relation of contiguity between mother and fetus, and there is absolutely no communication between the nervous systems of the two. Dr. E. T. Shelby, of Atchison, Kan., whom we have quoted in the above, says: "Cases of congenital deformity which should receive careful scientific investigation afford only an excuse for idle speculation concerning the kind of mental processes the mother must have experienced in order to produce a so-called fetal accident."—Ed. Charlotte Medical Journal, March, 1907.

